# Mutational screening of GDAP1 in dysphonia associated with Charcot-Marie-Tooth disease: clinical insights and phenotypic effects

**DOI:** 10.1186/s43141-023-00568-9

**Published:** 2023-11-15

**Authors:** Uzma Manzoor, Awais Ali, S. Luqman Ali, Omneya Abdelkarem, Sumaira Kanwal, Saqer S. Alotaibi, Alaa Baazeem, Aliya Baiduissenova, Ayaz Yktiyarov, Azraida Hajar, Abay Olzhabay

**Affiliations:** 1 Department of Clinical Biochemistry, COMSATS University Islamabad, Sahiwal Campus, Sahiwal, Pakistan; 2 Department of Biochemistry, Abdul wali Khan University Mardan, Mardan, 23200 Pakistan; 3 Department of Chemical Pathology, Medical Research Institute, Alexandria University, Alexandria, Egypt; 4 Department of Biotechnology, College of Science, Taif University, P.O.Box 11099, 21944 Taif, Saudi Arabia; 5 Department of Biology, College of Science, Taif University, P.O. Box 11099, 21944 Taif, Saudi Arabia; 6 Department of Microbiology and Virology, Astana Medical University, Astana City, 010000 Kazakhstan; 7 Department of Biology, Faculty of Sciences Semlalia, Cadi Ayyad University, Marrakech, Morocco; 8 Department of Otorhinolaryngology, Astana Medical University, Astana City, 010000 Kazakhstan

**Keywords:** Charcot-Marie-Tooth disease, Axonal CMT 2, Vocal cord dysphonia, GDAP1 mutations

## Abstract

**Introduction:**

Mutations in GDAP1 (Ganglioside-induced differentiation-associated protein 1) gene are linked to Charcot-Marie-Tooth disease (CMT), a Heterogenous group of disorders with multiple phenotypes, characterized by peripheral nerve dysfunction that can lead to vocal cord paralysis and diaphragmatic dysfunction.

**Main body:**

All three affected children of this chosen family have manifested the same clinical symptoms with progressive weakness, mild sensory impairment, and absent tendon reflexes in their early years. Electrodiagnostic analysis displayed an axonal type of neuropathy in affected patients. Sequencing of the GDAP1 gene was requested for all members of the family. Diagnostic assessments included pulmonary and vocal cord function tests, as well as phrenic and peripheral nerve conduction studies. Pathogenicity of GDAP1 variant p.Pro419Leu with axonal CMT2 and autosomal recessive inheritance was confirmed *via* in silico analysis. Patients with GDAP1 mutations showed dysphonia, speech difficulties, and the characteristic symptoms of CMT. The severity of symptoms correlated with the presence of a type of GDAP1 mutation. Patients with normal vocal cords and pulmonary function exhibited milder symptoms compared to those with GDAP1 mutations. Our study provides clinical insights into the phenotypic effects of GDAP1 mutations in CMT patients. The findings highlight the adverse clinical course and severe disability associated with GDAP1 mutations, including weak limb and laryngeal muscles.

**Conclusion:**

Patients with GDAP1 mutations and autosomal recessive neuropathy present with dysphonia and require interventions such as surgery, braces, physical therapy, and exercise. Early diagnosis and comprehensive clinical evaluations are crucial for managing CMT patients with GDAP1 mutations.

## Background

Charcot–Marie–Tooth disease is named after 3 researchers who described neuropathies in the late 1800s [1, 2]. CMT is a specific heterogeneous group of genetically sensory and motor peripheral neuropathies resulting from demyelination, axonal dysfunction, or both. It is the most common genetic neuropathy and most prevalent neuromuscular disease in children [3]. The progress of CMT is linked to the hereditary pattern, whereby the autosomal recessive form has an earlier onset and more severe symptoms than the autosomal dominant form.

CMT is a rare disease with a prevalence rate of 10–30 per 100,000 people, [4], it is rarely observed in Pakistan. Other community-based studies like that conducted in Egypt and included 42.223 individuals, identified five patients with CMT phenotypes, representing an estimated prevalence of 12/100,000 which describes the variations based on geographic origin [5].

Traditional classification of CMT (e.g., CMT1, CMT2, and DI-CMT [dominant intermediate]) was based on both peripheral neuropathy type as determined by nerve conduction velocity (NCV) and mode of inheritance as determined by family history. As understanding of the genetic basis of CMT gradually increased, letters in alphabetic order were assigned to the CMT type to represent the gene involved (e.g., CMT1A) [6]. As more genes causing CMT were identified and as the overlap of neuropathy phenotypes and modes of inheritance became apparent, the above alphanumeric classification system proved unwieldy and inadequate. In 2018, Magy et al. (2018) proposed a gene-based classification of inherited neuropathies An additional advantage of this classification system is that an individual’s findings can be described in terms of mode of inheritance, neuropathy type, and gene [7].

Clinically, patients with CMT have been categorized into three main groups based on nerve conduction velocities and nerve biopsy results. CMT Type 1A is the most common form of CMT accounting for approximately 60% of those with a genetic diagnosis [8]. Demyelinating CMT1 is characterized by a very slow motor nerve conduction velocity (MNCV) of 38 m/s. Occasionally, CMT2 cases exhibit almost normal MNCV, and an intermediate phenotype shows MCV between 30 and 40 m/s along with neurogenic atrophy and sensory nerve sparing, as detected by electromyography [9–11]. Demyelinating CMT, resulting from the duplication of the PMP22 gene, accounts for two-thirds of all CMT cases, while the mutation spectrum of CMT2 is more diverse and includes variants in genes such as MFN2, MPZ, GJB1, and GDAP1, among others [12].

Recent advances in molecular genetics mainly the next generation sequencing have improved our understanding of the causative genes that are related to the pathophysiology of CMT. To the best of our knowledge, more than 120 gene mutations are identified to cause CMT [13]. Eleven genes including LMNA, GDAP1, PMP22, MTMR2, MTMR13, Cx32/GJB1, PRX, MPZ, FGD4/FRABIN, SH3TC2, and GARS have been associated with CMT in Africa. Interestingly, five novel pathogenic mutations in consanguineous Pakistani families with early onset CMT have been reported in four genes namely SH3TC2, HK1, REEP1, and MFN2. Unfortunately, only a few studies have been performed to determine the genetic causes of CMT and related peripheral neuropathies worldwide.

Ganglioside-induced differentiation-associated protein 1 (GDAP1) is an integral mitochondrial outer membrane (MOM) protein, and the GDAP1 gene is one of the most prevalent in CMT-related missense mutations [8–10]. Both autosomal dominant and recessive modes of inheritance are found, resulting in either autosomal recessive, or dominant demyelinating CMT4, autosomal dominant axonal CMT2, or intermediate CMTRIA types of CMT, with varying phenotype severity.

GDAP1 mutations are particularly rare in Asia, with frequencies ranging from 0.6 to 2.37% among CMT patients in Japan and China [14–16]. In contrast, higher frequencies of GDAP1 mutations, approximately 7–14%, have been observed in CMT patients in Europe [17–21].

GDAP1 is localized in the outer mitochondrial membrane, it consists of six exons spanning a total length of 14 kb, located on chromosome 8q21. Amplification of the GDAP1 gene reveals different phenotypes of GDAP1 mutations associated with distinct diseases [22]. GDAP1 plays a crucial role in regulating mitochondrial functions and calcium homeostasis [23, 24]. While the GDAP1 gene encodes a protein of 358 amino acids belonging to a glutathione S-transferase (GST) enzyme subfamily, its exact cellular localization and functions are not yet fully understood, although it may impact axon–Schwann cell associations [10]. Numerous studies have investigated families from different ethnic backgrounds and described various GDAP1 gene mutations. Available data suggest that GDAP1 autosomal recessive (AR) inherited mutations lead to severe, early-onset neuropathy with axonal collapse and demyelinating variations. Vocal cord palsy can occasionally manifest in a chronic manner [6]. Most of these patients develop unilateral or bilateral vocal cord paresis, and diaphragmatic weakness in the latter stages of the disease [25]. It has been suggested that recessive mutations which cause truncating proteins develop a more severe phenotype, while missense mutations may be associated with a slightly milder course [26].

Changes in the sequence of the GDAP1 gene can cause several CMT phenotypes, including pes cavus, muscle degeneration, and hammered toes [27]. The inheritance pattern of CMT2K, associated with GDAP1, can vary significantly. In certain cases, mutations in the GDAP1 transporter, particularly the p.R120W variant, can have adverse effects, and their expression may be genetically modified by the junctophilin 1 gene [28]. Different types of mutations in the GDAP1 gene can lead to CMT 2K, which exhibits an autosomal recessive pattern of inheritance.

Vocal cord palsy and diaphragmatic dysfunction are rare and not specific to any particular type of CMT. These manifestations are characteristic of axonal CMT2C, linked to chromosome 12q23–24 [29]. Affected families have been reported to have demyelinating CMT4A [25], or axonal CMT (ARCMT2) diseases [30], while CMT has been associated with GDAP1. In both cases, the disease typically manifests in early infancy with phenotypes such as distal limb weakness, which progresses proximally and leads to severe disability. In some instances, nerve conduction velocity cannot be measured due to distal muscle atrophy and lack of response [6]. GDAP1 gene mutations associated with different inherited neuropathies exhibit a variable phenotypic spectrum [31].

## Materials and methods

### Sampling

A family was carefully selected after observing various relevant symptoms among 70 families. Their family history was traced back four generations using a pedigree. A questionnaire was designed to collect information about their condition. Blood samples were collected from the selected patients and their relatives at the Pediatric Neurology Children’s Complex in Multan for genetic analysis. The goal was to screen for mutations in the GDAP1 gene and correlate it with the clinical phenotype. Among families in Multan with inherited vocal dysphonia, five families were chosen for further analysis. The proband and other affected family members from the chosen family were examined and the collected samples were analyzed.

#### Subject

**Fig. 1:**
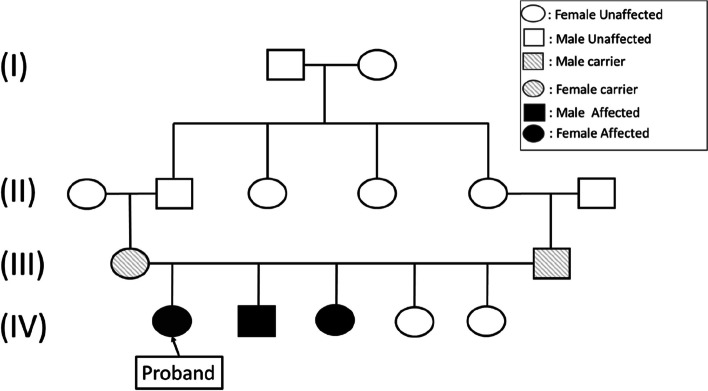
Pedigree of family suffering from CMT2 and inherited vocal cord dysphonia

A family of 7 members (Fig. 1) with CMT 2, living in Multan was enlisted from the population of Pakistan. Paternity had been assured by DNA typing using a multiplex short tandem repeats (STR) system (16 Power Plex) that allows genotyping of 15 microsatellites. (Madison, WI, Promega).

#### Clinical and electrophysiological data

Five families have been shortlisted. All individuals from 5 families were examined on the basis of symptoms. Clinical data spanning 15 months from the Medical Research Council was reviewed, as three patients from the selected family were undergoing medical treatment. This record included details about their bodily functions, such as breathing difficulties, stridor, sleep patterns, and walking problems. Electrophysiological records, magnetic resonance imaging, and nerve conduction velocities were also reviewed. A questionnaire was used to screen for stridor, voice disturbances, dysphagia, dyspnea at rest and after exercise, and snoring rate. Clinical symptoms have included effects on extremities such as the hands, lower legs, and feet. Myelinated somatic nerves have damaged myelin, so owing to abnormal connections within unusual myelin and axon, their NCV (nerve conduction velocities) are very slow. All individuals who were at risk of the disease have been assessed for dysphonia of voice, inability to speak aloud, sensory loss, reflexes, strength, muscle atrophy, scoliosis, change in voice, respiratory insufficiency, foot deformities, and changes in cranial nerves. Myopathy was done and muscle strength has been assessed by the standard MRC, i.e., Medical Research Council. The neuropathy score for CMT was used to assess neurological dysfunction [32]. Disability score via FDS and FSS scores was checked up to a 9-point scale, to observe the severity of fatigue in patients. Weakness occurs at distal parts with the furthest ends of long nerves due to disrupted interactions.

#### Electrophysiological study and nerve conduction velocities (NCV)

All three patients, who were at high risk in their entire family, reported neuropathy or complaints of neuropathy. A nerve conduction study had been verified by surface electrodes. Distal latency, CMAP (amplitudes for compound muscle action potentials), and conduction velocity from peroneal, median, tibial, ulnar, and axillary nerves were recorded by using traditional methods. CMAP and DL (distal latency) for the diaphragm have been measured using phrenic nerve stimulation in the neck region [10]. Sensory nerve action potential had been measured orthodromically. Needle myography was done with the proximal and distal muscles of the upper and lower limbs. Magnetic resonance imaging (MRI) were obtained using 1.5-T MRI system (Kim, H.S., et am 2021) and the neuropathy score was recorded after the whole analysis.

In CMT1A patients, progression is slow, and nerve conduction velocities are slow as well. The NCV of the intercostal and phrenic nerves is also slow, but comparatively short because they are not long, so they rarely develop breathing impairment. The same is true of CMTX.

#### Assessment of the vocal cords and phonation dysfunction studies

Laryngoscopy was done by an ENT physician using a flexible Laryngoscope, Vocal cord positions were assessed and reviewed for their results during phonation and inspiration. During respiration, the normal vocal cord separation is ~ 13.5 mm.

Phonation was checked and screened by observing vocal cords by a speech-language pathologist. Vocal oscillations and disturbance in voice have been checked. This study has included fundamental frequency, jitter, Lyapunov coefficient, estimated subglottic pressure, oral airflow intensity, signal-to-noise ratio, vocal range, and maximum phonation time. Phonation and inspiration. During respiration, the normal vocal cord separation is ~ 13.5 mm.

### Pulmonary dysfunction tests

Tests for pulmonary functions were performed by pulmonologists, such as expiratory as well as inspiratory pressures by flow-volume loop, and static lung volume were done using a Collins G II Plus spirometer (Collins, MA, USA). Arterial blood gases, an X-ray, and polygraphy for all patients were performed. Lung volume had been measured by the Collins BO-XII plethysmograph using reference values from the European guideline [33]. Respiratory polygraphy was performed at the bedside of patients during sleep and recorded heart rate, number of apneas and hypopneas per hour, and oxygen saturation were recorded.

#### Inclusion

We have searched for GDAP1 mutations systematically in all cases. There were 3 patients with GDAP1 mutations who were offered the opportunity to participate in this study. The pedigree is shown in Fig. 1, and it appears to be autosomal recessive. All parents in this pedigree were seen as unaffected, having only one consanguineous marriage.

#### Exclusion

The proband tested negative for CMT1A. PMP22, MPZ, and GJB1 genes were selected for mutation screening of the peripheral myelin genes. Mutations for other genes such as SEPT9, MPD2, MATR3, and MYH14 were excluded by observing phenotypes independently. MYH14 shows the phenotype of deafness, so it was excluded. GDAP1 was included, showing a particular phenotype of vocal dysphonia.

### DNA extraction

Genomic DNA samples have been extracted from all patients' venous blood by conventional processes. Purification was done by incubating DNA for 5 min at 56 °C in a thermocycler mix with agitation at full speed ~ 1400 rph, (NEB #T3010), using a SALSA MLPA Kits (MRC Holland).

### Primer designing for GDAP1 gene

Paired reliable primers (backward and forward) have been designed by using primer 3 software for the screening of genes. The primers have been selected based on their average length of 18–22 base pair (BP) and their 40–60% GC content. Appropriate primers have been applied to a group of patients to amplify GDAP1 exon and intron boundaries.

### PCR amplification

Required PCR conditions are as follows, 95 °C for 3 min, 30 cycles of 95 °C for 30 s, 60 °C for 30 s, and 72 °C for 45 s, then 72 °C for 6 min, after it was held at 4 °C. Allelle-specific PCR double digestion has been used for blood samples to amplify six exons of GDAP1 using a primer.

### Characterization of variations in GDAP1 and sequence analysis

PCR products have been screened, from which DNA fragments have been isolated. Peaks have been analyzed. Sequencing of GDAP1 (Accession No-000008 Region: 74350383.74488872) has been performed to identify a single pathogenic variant. Numerous bioinformatics techniques, such as Chromas, have been used to analyze DNA. Both Polyphen 2 and SIFT have confirmed the missense pathogenic variant.

## Results

### Clinical description of the proband and other family members

In the current study, a chosen family consisted of 7 family members, comprising of parents and 5 children. This disorder affected a 12-year-old female proband and her siblings, a 10-year-old boy and an 8-year-old girl. All of them were found to harbor the GDAP1 mutations. Clinical symptoms appeared in the proband at the age of 5 when she developed the typical symptoms of pes cavus foot and muscular dystrophy of the extremities. She complained of vocal cord dysphonia until the age of 5. She was diagnosed with CMT type 2. Nerve conduction studies and electromyography confirmed type 2 CMT because it was reduced and the proband demonstrated a prolonged delayed response. The proband's NCV was 35 m/s, which is significantly lower than the normal range of 40–45 m/s. Moreover, her two siblings born premature were also affected and showed similar phenotypes and the same clinical symptoms but to a lesser extent. Parents did not report any of the described symptoms. They have consanguineous backgrounds on the paternal side. Additionally, there were no abnormalities in either parent on the neurological examination or the nerve conduction study.

Nerve conduction studies and electromyography confirmed type 2 CMT because it was reduced and the proband demonstrated a prolonged delayed response. The proband’s NCV was 35 m/s, which is significantly lower than the normal range of 40–45 m/s. Moreover, her two siblings were also affected and showed similar phenotypes and the same clinical symptoms but to a lesser extent. Parents did not report any of the described symptoms. They have consanguineous backgrounds on the paternal side. Additionally, there were no abnormalities in either parent on the neurological examination or the nerve conduction study. However, ancillary tests had not been performed.

### Charcot-Marie-Tooth (CMT) disease symptoms

In this case, this family was selected because of the appearance of CMT symptoms in the girl and her siblings, like pes cavus foot, hammered toes, and hypotonia, with additional symptoms of dysphonia. During follow-up, they showed respiratory insufficiency on exertion. All patients had used noninvasive ventilation and were sporadic.

### Inheritance pattern and pedigree analysis

Family history was checked for three generations, but the disease appeared in 3rd generation in three siblings (2 girls and 1 boy). This could be explained by the consanguineous marriage of ancestors in the near past. Expression of disease in girls in this generation showed the presence of an autosomal recessive inheritance pattern.

### Musculoskeletal affection and electrophysiological studies

**Fig. 2:**
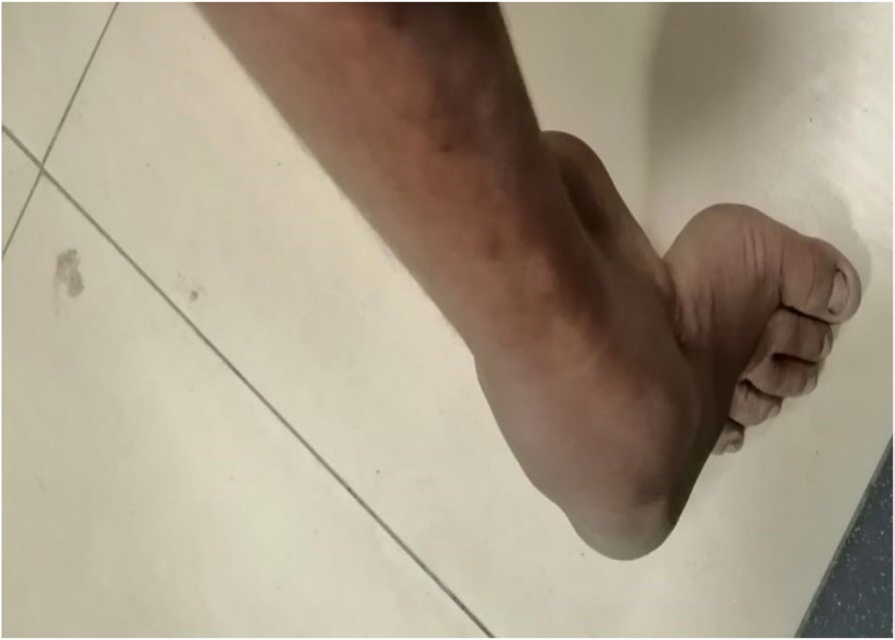
Proband showed pes cavus (high arch) and muscle atrophy at distal lower limbs

Three patients (two girls and one boy) aged 12, 10, and 8 years were included in this study. Clinical data has been condensed in Table 1. We presented these three patients with symptoms of congenital hypotonia and a delay in motor development. The most severe clinical abnormalities were lower limb weakness and muscle wasting in the distal extremities. All three patients had areflexia and other sensory abnormalities, which progressed to being chair-bound. The common presentation was difficulty in walking in early childhood. The Proband had difficulty walking and running when she was 2 years old, but she could climb stairs independently until the age of 5. Then she began to use foot orthoses at the same time. She began using crutches at the age of ten due to the progression of muscle weakness in her extremities. Clinical investigation had shown lower arm and lower leg atrophy, along with atrophy of hand muscles, as well as areflexia. The hand muscles and lower arm muscles were particularly weak. At the lower extremities, there were sensory impairments. Moreover, pain and touch sensations at distal ends were lost asymmetrically. The pes cavus was found to be the main symptom (Fig. 2).

**Table 1:** Clinical and electrophysiological data of chosen family members with type 2 axonal dysphonia

Chosen family	Age (years)	Onset age (years)	Type	NCV at distal Lower limbs	FDS (no of days/week	Associated disorder	CMAP and SNAP	Biopsy of sural nerve
Fat her		Nil	Nil	Nil	Nil	No clinical symptoms	Nil	Nil
Mother		Nil	Nil	Nil	Nil	No clinical symptoms	Nil	Nil
Proband	12	5	Type 2 axonal	35 m/s	2	Vocal cord dysphonia	Low amplitude	Pseudo bulb
3	10	5	Type 2 axonal	> 35 m/s	2	Vocal cord dysphonia	Low amplitude	Pseudo bulb
4	8	5	Type 2 axonal	> 35 m/s	2	Vocal cord dysphonia	Low amplitude	Pseudo bulb
5	1	Nil	Nil	Nil	Nil	Nil	Nil	Nil
6	2	Nil	Nil	Nil	Nil	Yet not Clinical symptoms	Nil	Nil

These patients, who proved to have AR (autosomal recessive) inherited mutations, had suffered severe neuropathy with early onset (0–12 years), leading to disability. All of these AR patients had major symptoms such as pes cavus and dysphonia. Respiratory malfunction was also observed, and sometimes patients needed non-invasive ventilation. Although patients were sporadic, intra-familial variability had not been seen. Among all AR patients, reduced SNAP (sensory nerve amplitude potential) as well as CMAP were clearly marked, as shown by unexcitable nerves, which included the sural and peroneal nerves. Reflex strength had shown 1+ (a slight response). Severity in the nerves of the lower limbs was shown by electrophysiological findings. EMG revealed re-innervation and de-innervation in the tibial muscles. Nerve conduction velocities of the proband were shown to be reduced, while measurements of conduction velocities of proximal muscles and MNCV were shown to be in the normal range, which proved axonal neuropathy.

The severity of the disease was emphasized by describing the progression of symptoms. Abnormal latencies were revealed by EMG. All patients were subjected to a series of electrophysiological studies at this early age. The patients did not show a distal motor response. There were abundant groups of small myelinated fibers. Myelin sheath was seen to be compact and alteration was not observed. There was a formation of crescent-shaped, concentric Schwann cells, mostly around small myelinated fiber clusters. The ‘pseudobulbs’ typical structures, considered to differentiate it from onion bulbs, are related to well-described demyelinating neuropathies [27]. The pathological picture had been precise as the progression of axonal deterioration but with vigorous non-effective axonal regeneration.

This disease was diagnosed as axonal CMT and we had taken into account the important fact that myelination has not been fully mature at this early age. That information was shown by a biopsy record of sural nerves. Axillary nerve latency was seen to be normal in all siblings. The clinical record and subsequent study showed low amplitudes of CMAPs and a lack of responses at 5 years of age, with severe clinical course. SNAP had persisted longer than CMAP, although it was abnormal which supports axonal CMT form.

### Pulmonary function tests interpretation

Evaluation of laryngeal and phrenic nerve responses was almost normal. Indirect laryngoscopy was used to examine laryngeal symptoms and the functioning of the respiratory system after patients reported voice changes, particularly when shouting or singing. Neither patient reported stridor or any other problems while sleeping, such as sputum production.

During respiration, the glottis remains open and is kept closed at the time of phonation. A flexible laryngoscope had shown atrophic vocal cords in patients, showing dysphonia. By questioning the patients and their families, it was reported that they faced dyspnea during speaking and exertion (Table 1).

These patients snored, but none of them presented with a significant abnormality of the thoracic cage. It was revealed by chest X-ray that diaphragm elevation had been normal in all patients. Furthermore, patients’ volume of air, maximum expiratory and inspiratory pressures, and spirometry values demonstrated severe functional alterations ranging from mild (75% of predicted values) to severe (38% of predicted values). The MIP (maximum inspiratory pressure), that is, the muscle strength, was reduced to 575% of predicted values. Gas exchange was shown by arterial blood gases that were in the normal range. Respiratory polygraphy was used to measure apnea, hypnoea index, and sleep hours, which were reported by 415 (516) patients.

### Genetic analysis

**Fig. 3:**
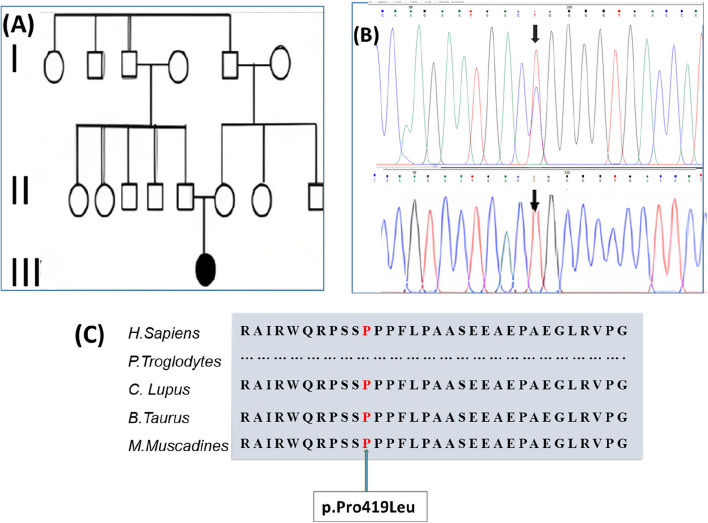
A Pedigree of the syndromic CMT Family (CMT-II). B Chromatogram showed the Proband with GDAP1 homozygous autosomal recessive mutation at p. Pro419Leu. C Conservation of amino acid at mutation position among several vertebrate species

A missense mutation in the GDAP1 gene c.692C>T was discovered in this family. This sequence change described in which proline amino acid is replaced with leucine amino acid at the 231 codons of the *GDAP1* gene resulting in a moderate physicochemical difference. Proline and leucine are both non-polar hydrophobic amino acids belonging to the same group. The family’s p.Pro419Leu pedigree as well as associated mutations are investigated, (shown in Fig. 2). We suspected type 2 CMT based on clinical data, nerve conduction studies, and family history, which revealed an autosomal recessive inheritance pattern. DNA testing revealed that the proband and her two siblings had a specific homozygous variation in the GDAP1 gene (Fig. 3). The proband, along with her two affected siblings, were carrying the specific missense variant p.Pro419Leu. The p.Pro419Leu variant is most likely a homozygous pathogenic variant. This variant was first described in a 9-year-old Puerto Rican patient without a family history of hereditary sensory motor neuropathy. This study enabled us to find a relationship between phenotype and genotype and, due to the GDAP1 AR mutation, symptoms like dysphonia (vocal dysphonia) and abnormalities in respiration. We found no patients with AR-inherited alterations in clinical differences based on mutation type or localization in this study. There was no clear difference with a missense mutation in onset age to wheelchair nor any severity score of truncating mutations among all patients, and no prominent and severe clinical variability was observed. The child’s parents were also tested, and the results were unremarkable for this pathogenic variant of the GDAP1 mutation gene without clinical manifestation of the neuropathy. As a result, a three-generation pedigree was obtained. Upon review, apart from the 2 siblings, there were no relatives with similar clinical presentations

## Discussion

Identification of a specific genetic cause of CMT hereditary neuropathy is a real challenge. Gene-targeted testing requires the clinician to hypothesize which gene(s) are likely involved. However, if there is an overlap of neuropathy phenotypes and modes of inheritance, a multigene panel that includes the most commonly involved genes is recommended. Comprehensive exome sequencing may be considered as the final option if both single gene testing or multigene panel fail to identify the exact genetic cause. Here based on the clinical presentation and the neurological examination in the context of inheritance raised the index of suspicion towards a particular type of CMT that needs confirmation with molecular genetic testing.

The main objective of this study was to screen GDAP1 in the selected family to confirm the presence of Charcot-Marie-Tooth type 2 sequencing of the GDAP1 gene expressed a homozygous p. Pro419Leu variant, which helped to confirm Charcot-Marie-Tooth type 2. It meant that their parents were known to be carriers, so they had not manifested any clinical symptoms of the disease. It was found that the c.692C > T (p.Pro231Leu) variant in exon 5 of the *GDAP1* gene is the most frequent pathogenic variant in the Old Order Amish population. DAP1, which belongs to the GST family (i.e., Glutathione-S-transferase subfamily), has two domains: GST-N and GST-C, (a-loop) 2 alpha-helical loops, (HD1), a domain of C-proximal hydrophobic which is critical for GDAP1 mutation, responsible for induced mitochondrial as well as peroxisomal fission, and (TMD) domain of C-terminal. For CMT pathogenesis, more than 100 GDAP1 variants are known to be implicated. these mutations are mostly nonsense or missense.

The cell expression with trimmed GDAP1 proteins, because of the absence of domains C-terminal and transmembrane, containing amino acids (320–358) has been misdirected within cell cytoplasm as well as the nucleus, which signifies that such types could be nonfunctional [24]. By distinction, mutant protein, p.R282C GDAP1 mutant has a correct position within mitochondria but mitochondrial fission is induced by way of wild-type GDAP1 [34].

Although the exact molecular mechanism underlying the GDAP1 function remains unclear, several studies have explored its role in mitochondria physiology: morphology, function, and dynamics [35]. First, GDAP1 mutations can impair these mitochondrial functions through mitochondrial membrane potential reduction, ATP production changes, or a disbalance of their dynamics [23, 36, 37].

Second, GDAP1 may also interact with transport proteins involved in mitochondrial transport and movement. Therefore, disruption of this process could explain the axonal loss that can be seen in CMT patients carrying GDAP1 mutations [38]. Finally, the recently established relationship between GDAP1 and mitochondrial-associated membranes (MAMs) which supports the idea that GDAP1 mutations could affect the formation and functioning of the ER-mitochondria contacts [39]. This could affect the store-operated Ca2+ entry (SOCE) and calcium homeostasis, together with mitochondrial dynamics and transport. On the other hand, recent studies have shown that GDAP1 participates in membrane contact sites (MCSs) between the mitochondria and the lysosome, supporting the idea that GDAP1 enables the proper function of mitochondrial MCSs [40, 41]. Finally, it has been found that it also influences the structure and probably the function of the Golgi apparatus [35]. Additionally, functional studies have characterized the phenotype derived from GDAP1 mutations. These include the alteration of mitochondrial fission-fusion events, changes in mitochondrial distribution, impairment of the mitochondrial membrane potential, increases in the concentration of reactive oxygen species, reductions in glutathione content, and alteration in the bioenergetics of mitochondria.

Additionally, it was found that GDAP1 plays an important role in the interaction between Schwann cells and axon. Interruption of this interaction may cause either axonal degeneration or demyelination in the peripheral nerve. Mutated *GDAP1* might prevent the correct catalyzing S-conjugation of reduced GSH, resulting in progressive attenuation of both axons and Schwann cells [42]. Genetic heterogeneity in CMT ensures the same phenotype of hereditary peripheral neuropathy associated with different mutations of numerous genes.

CMT involving the GDAP1 mutations generally leads to aggressive disorders appearing in infancy or early childhood [6]. The clinical presentation of our patients was in agreement with most cases, the disease leads to disability such as wheelchair dependency before the second to third decade of life. In some cases, the presence of vocal cord paresis and diaphragmatic paralysis suggests the clinical progression of the disease [26]. Compared to the dominant forms, autosomal recessive forms of CMT that involve GDAP1 mutations are more severe and less common in the general population but account for the vast majority of CMT phenotypes in communities with a high prevalence of consanguinity which was evident in our study.

The preliminary symptom in several cases was voice alteration, which was followed by the next reported atrophy of the hand muscles. The clinical picture in these patients, like ours, was length-associated neuropathy that primarily affected the lower limbs rather than the upper limbs. While CMT conditions accompanied by vocal cord paresis were mentioned in those who were not genetically characterized. In all patients with GDAP1-associated neuropathy [CMT4A], [17, 43–47].

Among CMT-associated complications, vocal cord paralysis remains an underestimated finding. Vocal cord paralysis has been reported as an early, severe, and frequent symptom of GDAP1-associated neuropathy [CMT4A], often followed by diaphragmatic dysfunction [8–13], and in both AD and AR inheritance forms of CMT2A (mutations in the MFN2 gene) [14, 15]. Few cases are reported in axonal dominant CMT2C (mutations in the transient receptor potential cation channel, subfamily V, member 4; TRPV4) [16–18] and as a later involvement in a patient harboring a dominant heterozygous mutation of early growth response 2 (EGR2) gene [19].

Vocal cord palsy with GDAP1 neuropathy has been observed in patients with p.Q163X and p.S194X mutations. In this case, all mutations were linked with the involvement of vocal cords, except p.R282C. The less severe phenotypic patients with no participation of the vocal cords or diaphragm had progressed. The p.R282C mutation has been reported in one Turkish family and one of Croatian origin [48, 49], with the clinical course being less severe in these affected people with GDAP1 neuropathy.

Regarding CMT4B1, vocal cord paralysis was reported in one Italian patient, one English patient, and three Algerian siblings by Sabatelli, Tyson, and Houlden [50, 51] and Nouioua [52], respectively. All of the patients suffered from early-onset (1–2 years old) severe demyelinating neuropathy associated with respiratory difficulties. The Algerian siblings presented prominent chest deformities possibly secondary to breathing impairment. In the latter cases sequencing analysis revealed mutations located in exon 4 (c.G308A; p.Gly103Glu and c.331dupA; p.Arg111LysfsX24, respectively). Notably, laryngeal involvement was present in the early stages of the disease in all cases.

Their clinical profile had the same features as described in Moroccan families, stated by [53], those were known to be homozygous mutation, p.S194X in GDAP1, but our case had, somehow more severe course. The majority of Moroccan patients had been used to walking by a walking stick in the second decade of life while our cases had been wheelchair-bound.

Furthermore, vocal cord paresis could be unnoticed during the usual neurological assessment. Our cases, despite allelic heterogeneity, disease history, and clinical phenotypes of our axonal type CMT patients had been fairly homogenous, as shown in proband. The patient's pathology is dissimilar from others. Patient, for missense p.R282C mutation, was homozygous while others were homozygous for mutations, predicting truncated protein. Protein, i.e., GDAP1 which is positioned at (OMM) [42] has two domains, GST and trans.

It may be debated that the severe damaging effects may be because of nonfunctional mutations, rather than that of missense mutation of GDAP1 protein and its expression within the cell. Thus, our study shows the mutation of p.Pro419Leu mutations and its deleterious effects on nerve physiology. All three patients in our study had offered restrictive changes in respiratory working and diaphragm elevation.

It was demonstrated by chest X-ray as well as reduced CMAP of the phrenic nerve that respirational impairment was because of muscle weakness. The same features are reported in some other affected persons having CMT and diaphragm weakness [54, 55].

Normal blood gases had been observed in our patients and indicated the absence of hypoventilation in all. Four factors contributed to respiratory impairment in our patients. One patient had neither pulmonary abnormalities nor proximal upper limb weakness. Predictive factors for respiratory system involvement in this study the weakness of the proximal part of the upper limb and bilateral diaphragmatic paresis; obesity plays a significant role in determining severity. Our findings are consistent with previous findings that age and weakness of the proximal part of the upper limb are prognostic for pulmonary impairment [56, 57].

Obstructive sleep apnea syndrome was discovered in two of our patients. severe pharyngo-laryngeal neuropathy was the main cause of this obstructive form, it was known to be like the central form, which was related to the syndrome of obesity-hypoventilation. However, some studies have found a link between severe neuropathy and the syndrome of sleep apnea in CMT1 patients [55, 58], Establishing the specific cause of CMT hereditary neuropathy for a given individual involves obtaining a medical history and performing a physical examination to exclude disorders that differ from CMT as systemic disorders with neuropathy, other hereditary neuropathies, distal myopathies, hereditary sensory neuropathies (HSN) and hereditary sensory and autonomic neuropathies (HSAN), and acquired disorders. The complexity of interpreting genetic test results makes referral to specialized neurogenetics centers a necessity that needs to be considered by healthcare providers in order to achieve early diagnosis and improve the prognosis.

## Conclusion

*GDAP1* mutation needs to be considered as the probable cause of CMT in the assessment of pediatric patients with clinical and electrophysiological evidence of early onset axonal sensorimotor neuropathy. The NCV and EMG findings must be included as valuable tools in the clinical approach of the patient with Charcot-Marie-Tooth disease, recessive axonal type. Further studies for the identification of novel mutations in the GDAP1 associated with vocal cord paralysis and respiratory affection in different ethnic groups are highly recommended.

## Data Availability

The data that support the findings of this study are available in PubMed and Google Scholar, and the applicable resources are available in the public domain as mentioned in the review with references.
